# Real-world use of blinatumomab in adult patients with B-cell acute lymphoblastic leukemia in clinical practice: results from the NEUF study

**DOI:** 10.1038/s41408-022-00766-7

**Published:** 2023-01-04

**Authors:** Nicolas Boissel, Sabina Chiaretti, Cristina Papayannidis, Josep-Maria Ribera, Renato Bassan, Andrey N. Sokolov, Naufil Alam, Alessandra Brescianini, Isabella Pezzani, Georg Kreuzbauer, Gerhard Zugmaier, Robin Foà, Alessandro Rambaldi

**Affiliations:** 1 Division of Hematology, EA3518 Saint-Louis Institute for Research, Saint-Louis Hospital, Paris, France; 2 Hematology Department of Translational and Precision Medicine, “Sapienza” University, Rome, Italy; 3 IRCCS, Azienda Ospedaliero Universitaria di Bologna, Institute of Hematology “Seràgnoli”, Bologna, Italy; 4 Clinical Hematology Department, ICO-Hospital Germans Trias i Pujol, Josep Carreras Leukaemia Research Institute, Universitat Autònoma de Barcelona, Badalona, Spain; 5 Complex Operative Unit of Hematology, dell’Angelo Hospital and Santissimi Giovanni and Paolo Hospital, Mestre and Venice, Venezia-Mestre, Italy; 6 National Research Center for Hematology, Moscow, Russian Federation; 7 Amgen Ltd, Uxbridge, United Kingdom; 8 Amgen (Europe) GmbH, Rotkreuz, Switzerland; 9 Amgen Research (Munich) GmbH, Munich, Germany; 10 Department of Oncology and Haematology, University of Milan and Azienda Socio Sanitaria Territoriale Papa Giovanni XXIII, Bergamo, Italy

**Keywords:** Acute lymphocytic leukaemia, Cancer immunotherapy

## Abstract

This retrospective observational study (NEUF) included adult patients with B-cell acute lymphoblastic leukemia (B-cell ALL) who had received blinatumomab for the treatment of minimal residual disease-positive (MRD+) or relapsed/refractory (R/R) B-cell ALL via an expanded access program (EAP). Patients were eligible if blinatumomab was initiated via the EAP between January 2014 and June 2017. Patients were followed from blinatumomab initiation until death, entry into a clinical trial, the end of follow-up, or the end of the study period (December 31, 2017), whichever occurred first. Of the 249 adult patients included, 109 were MRD+ (83 Philadelphia chromosome-negative [Ph−] and 26 Philadelphia chromosome-positive [Ph+]) and 140 had a diagnosis of R/R B-cell ALL (106 Ph− and 34 Ph+). In the MRD+ group, within the first cycle of blinatumomab treatment, 93% (*n* = 49/53) of Ph− and 64% (*n* = 7/11) of Ph+ patients with evaluable MRD achieved an MRD response (MRD <0.01%). Median overall survival (OS) was not reached over a median follow-up time of 18.5 months (Ph−, 18.8 [range: 5.1–34.8] months; Ph+, 16.5 [range: 1.8–31.6] months). In the R/R group, within two cycles of blinatumomab, 51% of Ph− and 41% of Ph+ patients achieved complete hematologic remission (CR/CRh/CRi), and 83% of Ph− and 67% of Ph+ MRD-evaluable patients in CR/CRh/CRi achieved an MRD response. Median (95% confidence interval) OS was 12.2 (7.3–24.2) months in the R/R Ph− subgroup and 16.3 (5.3–not estimated) months in the R/R Ph+ subgroup. This large, real-world data set of adults with B-cell ALL treated with blinatumomab confirms efficacy outcomes from published studies.

## Introduction

Approximately 30–50% of patients with B-cell acute lymphoblastic leukemia (B-cell ALL) in complete hematologic remission (CR/CRh/CRi) exhibit post-induction minimal residual disease (MRD) persistence, which indicates resistance to standard chemotherapy [1]. The presence of MRD-positive (MRD+) disease is also associated with a high relapse rate and poor overall survival (OS) [1, 2], representing the most important risk factor for hematologic relapse in adult patients with B-cell ALL [3]. Patients with relapsed or refractory (R/R) acute lymphoblastic leukemia (ALL) have poor long-term survival and limited treatment options. MRD eradication is, therefore, the goal for patients with B-cell ALL; this highlights the importance of incorporating MRD assessment into routine clinical practice [3].

Blinatumomab is a bispecific T-cell engager (BiTE®) molecule that directs CD3-expressing T cells to CD19-expressing B cells, resulting in tumor cell lysis [4, 5]. The efficacy of blinatumomab in patients with B-cell ALL has been demonstrated in various studies. In a Phase 2 trial in patients with MRD+ B-cell ALL, 78% of patients receiving blinatumomab achieved a complete MRD response, which was associated with longer relapse-free survival (RFS) and OS compared with MRD nonresponders [2, 6]. In two single-arm Phase 2 studies, blinatumomab induced hematologic remission in heavily pretreated patients with R/R Philadelphia chromosome-negative (Ph−) B-cell ALL [7, 8]. In a Phase 3 trial, treatment with blinatumomab resulted in longer median OS than standard chemotherapy [9]. MRD results were also analyzed in this patient population and showed longer OS for MRD responders than for MRD nonresponders; the difference was greater in first salvage than in second or later salvage [10, 11]. In a Phase 2 trial in patients with R/R Ph+ B-cell ALL, 36% of patients achieved complete remission (CR), of whom 88% attained MRD negativity [12].

Evidence of real-world use of blinatumomab to date is based on data from a compassionate use program in France, an ad hoc survey in Italy, and a retrospective cohort study in the USA [13–15]. These data sets demonstrated responses comparable to those reported in clinical trials [2, 9].

Before country-specific approval, blinatumomab was made available to patients who met prespecified criteria via an expanded access program (EAP). The NEUF study was a retrospective observational study including both adult and pediatric patients receiving blinatumomab treatment via an EAP.

## Materials and methods

### Objective

To describe the clinical characteristics and treatment patterns of adult patients with B-cell ALL who received blinatumomab in the EAP, and in pre-identified clinically relevant subgroups (i.e., MRD+ Ph−/Ph+ and R/R Ph−/Ph+ subgroups).

### Patient selection

As described previously, patients treated with blinatumomab in the EAP between January 1, 2014, and June 30, 2017, in France, Italy, the Russian Federation, Spain, and the UK were eligible for inclusion [16]; the sites with the largest number of requests were invited to participate in the NEUF study. Patients were not eligible for inclusion in the study if informed consent was not provided (per local regulations) or if their medical records were not available. Adverse events were reported separately and according to local regulations, as detailed previously [16].

### Outcome measures

CR was defined as ≤5% blasts in the bone marrow, no evidence of extramedullary disease, and full recovery of peripheral blood counts (platelets >100 × 10^9^/L and absolute neutrophil count >1 × 10^9^/L) [9, 16, 17]. CR with partial recovery of peripheral blood counts (CRh) was defined as CR, but with partial recovery of peripheral blood counts (platelets >50 × 10^9^/L and absolute neutrophil count >0.5 × 10^9^/L). CR with incomplete recovery of peripheral blood counts (CRi) was defined as CR, but with incomplete recovery of peripheral blood counts (platelets >100 × 10^9^/L or absolute neutrophil count >1 × 10^9^/L) among patients with R/R B-cell ALL at blinatumomab initiation.

MRD assessment was undertaken as per local clinical practice using a technique with a quantifiable sensitivity down to 10^−4^ assessed by flow cytometry and polymerase chain reaction. Standardized protocols [18–21] were available for use in routine clinical practice in the participating countries, and technique choice was at the discretion of each investigator/site. MRD status was abstracted from each patient’s medical record, and available MRD data were analyzed irrespective of the technique used. Patients categorized as having a complete MRD response were those in CR/CRh/CRi with undetectable MRD within the first cycle or the first two cycles of blinatumomab treatment, depending on how many cycles were required. MRD response was defined as CR/CRh/CRi with low-level nonquantifiable MRD (<10^−4^/0.01%). Patients with persistent MRD were those without an MRD response or complete MRD response (quantifiable MRD, ≥10^−4^/0.01%). Patients with MRD relapse were those in CR/CRh/CRi and who had a prior complete MRD response or an MRD response that was subsequently lost (quantifiable MRD).

In the MRD+ group, disease-free survival (DFS) was evaluated and described as the time from blinatumomab initiation to the date of relapse or death in remission, whichever occurred first.

In the R/R B-cell ALL group, relapse was defined as >5% blasts in the bone marrow or extramedullary relapse after documented CR/CRh/CRi. RFS was estimated for the R/R B-cell ALL group and defined as the time from achievement of CR/CRh/CRi (best response within the first two cycles) to the date of relapse or death in remission, whichever occurred first. In both groups, OS was defined as the time from initiation of blinatumomab treatment until death from any cause.

Enrolled patients were observed from blinatumomab initiation until death, entry into a clinical trial, the end of follow-up, or the end of the study (December 31, 2017), whichever occurred first.

The study was approved by the appropriate local institutional review boards/independent ethics committees and was conducted in accordance with the principles of the Declaration of Helsinki and Good Clinical Practice guidelines issued by the International Conference of Harmonisation.

### Statistical analyses

Analyses were descriptive and no formal hypothesis was tested [16]. Time-to-event analysis (e.g., for OS, DFS, and RFS) was undertaken using Kaplan–Meier (KM) methodology and using inverse KM estimates to calculate follow-up time. Mortality following allogeneic hematopoietic stem cell transplantation (HSCT) was evaluated as mortality not due to disease relapse, treating relapse and death due to undocumented relapse as competing risks. Mortality estimates were calculated using cumulative incidence function analysis. To address immortal time bias (i.e., relapse is not possible prior to response), landmark analyses were undertaken, comparing OS between patients who responded to blinatumomab treatment within the first two cycles and those who did not. Any patient who did not report a response to blinatumomab treatment within two cycles (84 days) was classed as a nonresponder, even if they later had a response.

## Results

### Patient characteristics

Overall, 249 patients were included, of whom 109 were MRD+ (83 Ph− and 26 Ph+) and 140 had a diagnosis of R/R B-cell ALL (106 Ph− and 34 Ph+) (Table 1). All patients in the Ph+ subgroup had the *BCR-ABL1* translocation confirmed prior to blinatumomab initiation (Supplementary Table 1). Owing to missing diagnosis data, four additional patients were excluded from these analyses. In the MRD+ group, most of the patients (71%) had persistent MRD irrespective of the number of prior salvages and their Ph status. Patients who had no prior salvage therapy (CR1) represented 60% and 38% of the Ph− and Ph+ subgroups, respectively. The remaining patients (40% of Ph− and 62% of Ph+) had received at least one prior salvage therapy (CR2+). In the R/R subgroups, 42% of Ph− patients had no prior salvage (76% relapsed and 24% refractory) and 12% of Ph+ patients had no prior salvage (50% relapsed and 50% refractory).

**Table 1:** Baseline characteristics.

	MRD+ (*N* = 109)						R/R B-cell ALL (*N* = 140)			
Characteristics^a^	All MRD+ (*N* = 109)		Ph− (*N* = 83)		Ph+^a^ (*N* = 26)		R/R Ph− (*N* = 106)		R/R Ph+^a^ (*N* = 34)	
Sex, female, *n* (%)	45 (41.3)		39 (47.0)		6 (23.1)		50 (47.2)		16 (47.1)	
Age at blinatumomab initiation, years, median (IQR)	43.0 (27.0–55.0)		35.0 (24.0–56.0)		50.5 (43.0–55.0)		36.5 (24.0–52.0)		51.0 (37.0–64.0)	
Number of salvage therapies										
Median (IQR)	0 (0–1)		0 (0–1)		0 (0–2)		1 (0–2)		1 (1–2)	
Min., max.	0, 4		0, 4		0, 4		0, 5		0, 5	
Prior salvage therapy, *n* (%)	*n* = 77^b^	*n* = 32^c^	*n* = 57^b^	*n* = 26^c^	*n* = 20^b^	*n* = 6^c^	*n* = 64^d^	*n* = 42^e^	*n* = 20^d^	*n* = 14^e^
0	42 (54.5)	18 (56.2)	35 (61.4)	15 (57.7)	7 (35.0)	3 (50.0)	34 (53.1)	11 (26.2)	2 (10.0)	2 (14.3)
1	21 (27.3)	8 (25.0)	14 (24.6)	6 (23.1)	7 (35.0)	2 (33.3)	13 (20.3)	19 (45.2)	8 (40.0)	6 (42.9)
2+	14 (18.2)	6 (18.8)	8 (14.0)	5 (19.2)	6 (30.0)	1 (16.7)	17 (26.6)	12 (28.6)	10 (50.0)	6 (42.8)
Disease status at blinatumomab initiation, *n* (%)										
Full hematologic relapse	NE		NE		NE		64 (60.4)		20 (58.8)	
Refractory	NE		NE		NE		42 (39.6)		14 (41.2)	
Persistent MRD	77 (70.6)		57 (68.7)		20 (76.9)		NE		NE	
MRD relapse	32 (29.4)		26 (31.3)		6 (23.1)		NE		NE	
HSCT before blinatumomab initiation, *n* (%)	17 (15.6)		9 (10.8)		8 (30.8)		43 (40.6)		12 (35.3)	
Time between HSCT and initiation, months, median (IQR)	10.2 (3.8–24.9)		9.5 (3.8–21.3)		13.5 (5.4–36.7)		13.0 (7.2–20.0)		10.4 (7.1–20.6)	
Response before blinatumomab initiation, *n* (%)										
CR/CRh/CRi at frontline therapy	NE		NE		NE		84 (79.2)		25 (73.5)	
Blast count in the bone marrow at blinatumomab initiation, *n* (%)										
≤5	86 (91.5)		65 (91.5)		21 (91.3)		14 (14.6)		2 (7.1)	
>5 and <10	2 (2.1)		2 (2.8)		0 (0.0)		5 (5.2)		0 (0.0)	
≥10 and <50	2 (2.1)		2 (2.8)		0 (0.0)		33 (34.4)		12 (42.9)	
≥50	4 (4.3)		2 (2.8)		2 (8.7)		44 (45.8)		14 (50.0)	
Unknown	15 (NA)		12 (NA)		3 (NA)		10 (NA)		6 (NA)	
Extramedullary involvement, *n* (%)										
Yes	NA		NA		NA		20 (19.2)^f^		5 (14.7)	
Central nervous system	NA		NA		NA		4 (3.8)		5 (14.7)	
Testis	NA		NA		NA		4 (3.8)		0 (0.0)	
Other	NA		NA		NA		12 (11.5)		0 (0.0)	
No	NA		NA		NA		84 (80.8)		—	
Unknown	NA		NA		NA		2 (NA)		—	

^a^Cytogenetics of patients with Ph+ B-cell ALL are presented in Supplementary Table 1.

^b^For the subset of patients who have persistent MRD.

^c^For the subset of patients who are in MRD relapse.

^d^For the subset of patients who are relapsed.

^e^For the subset of patients who are refractory.

^f^Response to blinatumomab was examined in a subgroup of patients with extramedullary involvement.

*B-cell ALL* B-cell acute lymphoblastic leukemia, *CR* complete remission with full recovery of peripheral blood counts, *CRh* complete remission with partial recovery of peripheral blood counts, *CRi* complete remission with incomplete recovery of peripheral blood counts, *HSCT* hematopoietic stem cell transplantation, *IQR* interquartile range, *max*. maximum, *min*. minimum, *MRD* minimal residual disease, *MRD*+ MRD-positive, *NA* not applicable, *NE* not estimated, *Ph*− Philadelphia chromosome-negative, *Ph*+ Philadelphia chromosome-positive, *R/R* relapsed/refractory.

### Concurrent therapies during blinatumomab treatment

Within each group, patients received a median of two cycles of blinatumomab. In the MRD+ group, four patients (two Ph− and two Ph+) were treated with donor lymphocyte infusion (DLI). In the R/R B-cell ALL group, 13 patients (all Ph−) were treated with DLI. Concurrent use of tyrosine kinase inhibitors (TKIs) was documented for 12 patients in the MRD+ Ph+ subgroup and 14 patients in the R/R Ph+ subgroup. Use of TKIs is reported in Supplementary Fig. 1 and Supplementary Table 2, and comedications of interest are summarized in Supplementary Table 3.

### Response to blinatumomab treatment

#### MRD+ group

**Fig. 1:**
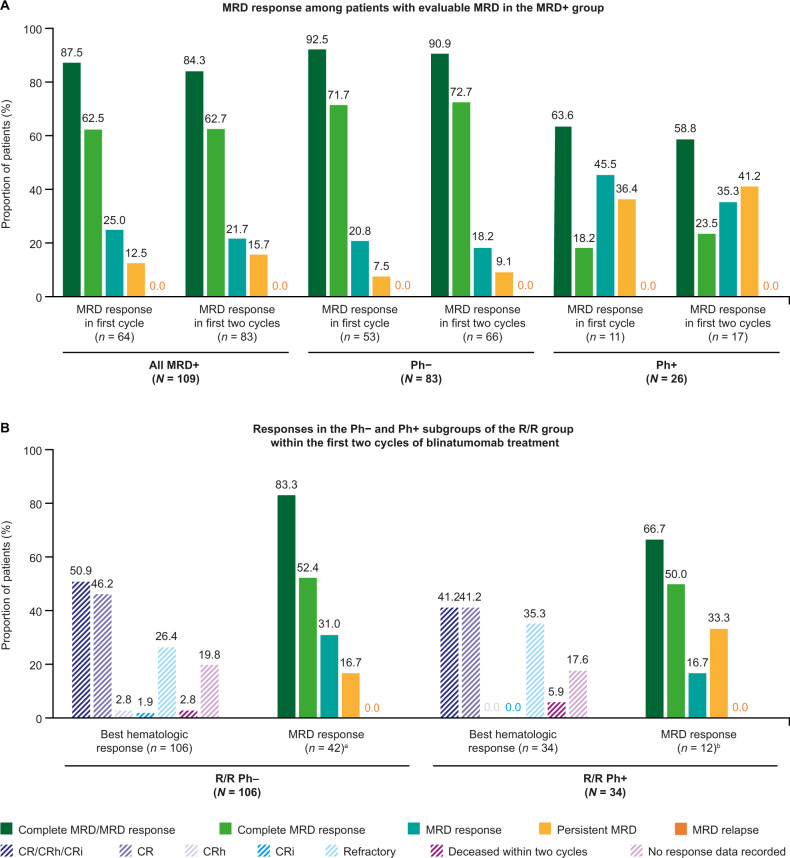
Response to blinatumomab treatment. **A** MRD response among patients with evaluable MRD in the MRD+ group. **B** Responses in the Ph− and Ph+ subgroups of the R/R group within the first two cycles of blinatumomab treatment. ^a^In total, 106 adult R/R Ph− B-cell ALL patients were included in the study; 54 patients achieved CR/CRh/CRi within the first two cycles of blinatumomab treatment. Of these patients, 42 had evaluable MRD at two cycles. ^b^In total, 34 adult R/R Ph+ B-cell ALL patients were included in the study; 14 patients achieved CR/CRh/CRi within the first two cycles of blinatumomab treatment. Of these patients, 12 had evaluable MRD at two cycles. *B-cell ALL* B-cell acute lymphoblastic leukemia, *CR* complete remission with full recovery of peripheral blood counts, *CRh* complete remission with partial recovery of peripheral blood counts, *CRi* complete remission with incomplete recovery of peripheral blood counts, *MRD* minimal residual disease, *MRD*+ MRD-positive, *Ph*− Philadelphia chromosome-negative, *Ph*+ Philadelphia chromosome-positive, *R/R* relapsed/refractory.

In patients with evaluable MRD (*n* = 64), 88% (*n* = 56) achieved an overall MRD response (either a complete MRD response or an MRD response) within the first cycle of blinatumomab. Among the 83 patients who were evaluated for MRD within two cycles of blinatumomab, 84% (*n* = 70) had an overall MRD response, representing 91% and 59% of patients in the Ph− and Ph+ subgroups, respectively (Fig. 1A). The overall MRD response did not differ by CR1 or CR2+ status in both the Ph− (88% and 91%, respectively) and the Ph+ subgroup (57% and 56%, respectively).

#### R/R group

Within the first two cycles of blinatumomab, 54 patients (51%) in the R/R Ph− subgroup achieved CR/CRh/CRi, of whom 91% (49/54) achieved CR (Fig. 1B). Among these 54 patients, 42 had an evaluable MRD assessment within the first two cycles of blinatumomab and 83% (*n* = 35) achieved an overall MRD response (22 had a complete MRD response [52%]; 13 had an MRD response [31%]) (Fig. 1B).

Within the first two cycles of blinatumomab, 14 patients (41%) in the R/R Ph+ subgroup achieved CR/CRh/CRi and all patients achieved CR (Fig. 1B). Of these patients, 12 had an evaluable MRD assessment (Fig. 1B), and eight of these (67%) achieved either a complete MRD response (50%; *n* = 6) or an MRD response (17%; *n* = 2).

#### Patients with extramedullary involvement

In the R/R group, of the 20 Ph− patients with extramedullary involvement, 10 patients (50%) achieved complete hematologic remission with five of these achieving an MRD response. Among the five Ph+ patients with extramedullary involvement, three achieved complete hematologic remission, of whom two achieved an MRD response.

#### Patients with DLI

In the MRD+ group, two of the four patients treated with DLI had evaluable MRD and both (one Ph− and one Ph+) achieved an MRD response. In the Ph− subgroup, 69% (*n* = 9) of patients who received a DLI achieved CR/CRh/CRi following two cycles of blinatumomab, and five of the six evaluable patients (83%) achieved an overall MRD response.

### Survival outcomes

#### OS and DFS in the MRD+ group

**Fig. 2:**
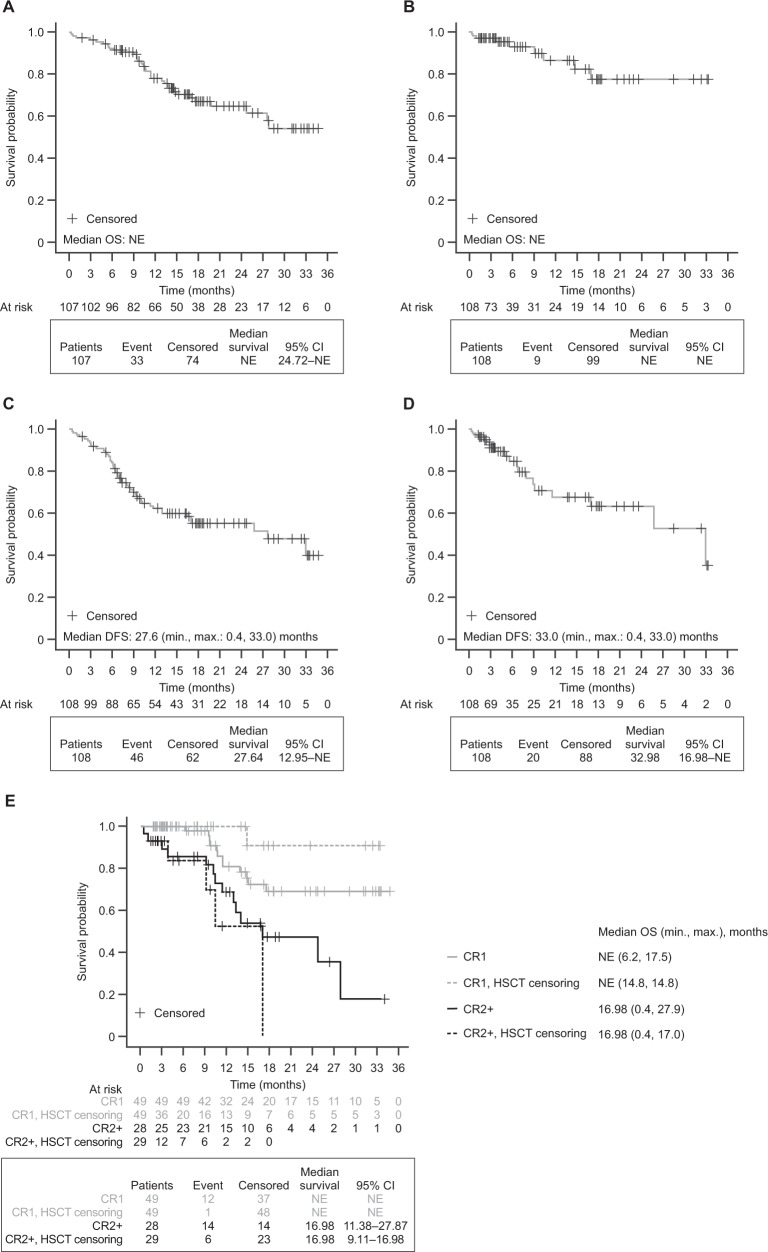
KM analysis of OS and DFS in the MRD+ group. **A** OS. **B** OS with additional censoring at the time of HSCT. **C** DFS. **D** DFS with additional censoring at the time of HSCT. **E** OS by CR1/CR2+ status with or without additional censoring at the time of HSCT. In the OS analysis, two adult patients were excluded from the crude analysis and one patient was excluded from the HSCT-censored analysis because of missing data on the date of the event. In the DFS analysis, one patient was excluded because of missing data (dates). For OS and DFS, patients were censored if they were alive at the end of the study or they were lost to follow-up. *CI* confidence interval, *CR1* defined patients who reached complete remission after first induction therapy*, CR2*+ defined patients who reached complete remission after one or more salvage therapies for relapsed or refractory disease, *DFS* disease-free survival, *HSCT* hematopoietic stem cell transplantation, *KM* Kaplan–Meier, *max*. maximum, *min*. minimum, *MRD*+ minimal residual disease-positive, *NE* not estimable, *OS* overall survival.

Median OS and median OS censored at the time of HSCT were not reached (Fig. 2A, B). Median follow-up was 18.5 months (Supplementary Table 4). Achieving an overall MRD response in the first two cycles of blinatumomab resulted in better KM estimates for OS at 24 months among MRD responders (71.5%) than among MRD nonresponders (57.1%; persistent MRD or MRD relapse) (Supplementary Fig. 2A). Similar results were found in a landmark analysis (Supplementary Table 5). Median (95% confidence interval [CI]) DFS was 27.6 (13.0–not estimated [NE]) months (Fig. 2C; Supplementary Table 6), and 33.0 (17.0–NE) months with additional censoring at the time of HSCT (Fig. 2D). DFS estimates at 24 months were better in MRD responders than in nonresponders (KM estimates: 61.2% of MRD responders vs 36.9% of MRD nonresponders; Supplementary Fig. 3).

In both Ph− and Ph+ subgroups, the KM estimates for OS and DFS at 24 months were consistent with those in the overall MRD+ group (Supplementary Fig. 2A; Supplementary Fig. 3; Supplementary Table 4; Supplementary Table 5). DFS was 25.7 months in the Ph− subgroup (median follow-up, 18.6 months) and was not reached in the Ph+ subgroup (median follow-up, 16.2 months) (Supplementary Table 6). In the Ph− subgroup, better outcomes were observed in the CR1 subgroup than in the CR2+ subgroup (median OS: NE versus 16.98 months [Fig. 2E]; median DFS: 32.98 versus 11.34 months). The KM estimates for OS and DFS at 24 months are shown in Supplementary Figs. 2A and 3.

#### OS and RFS in the R/R group

**Fig. 3:**
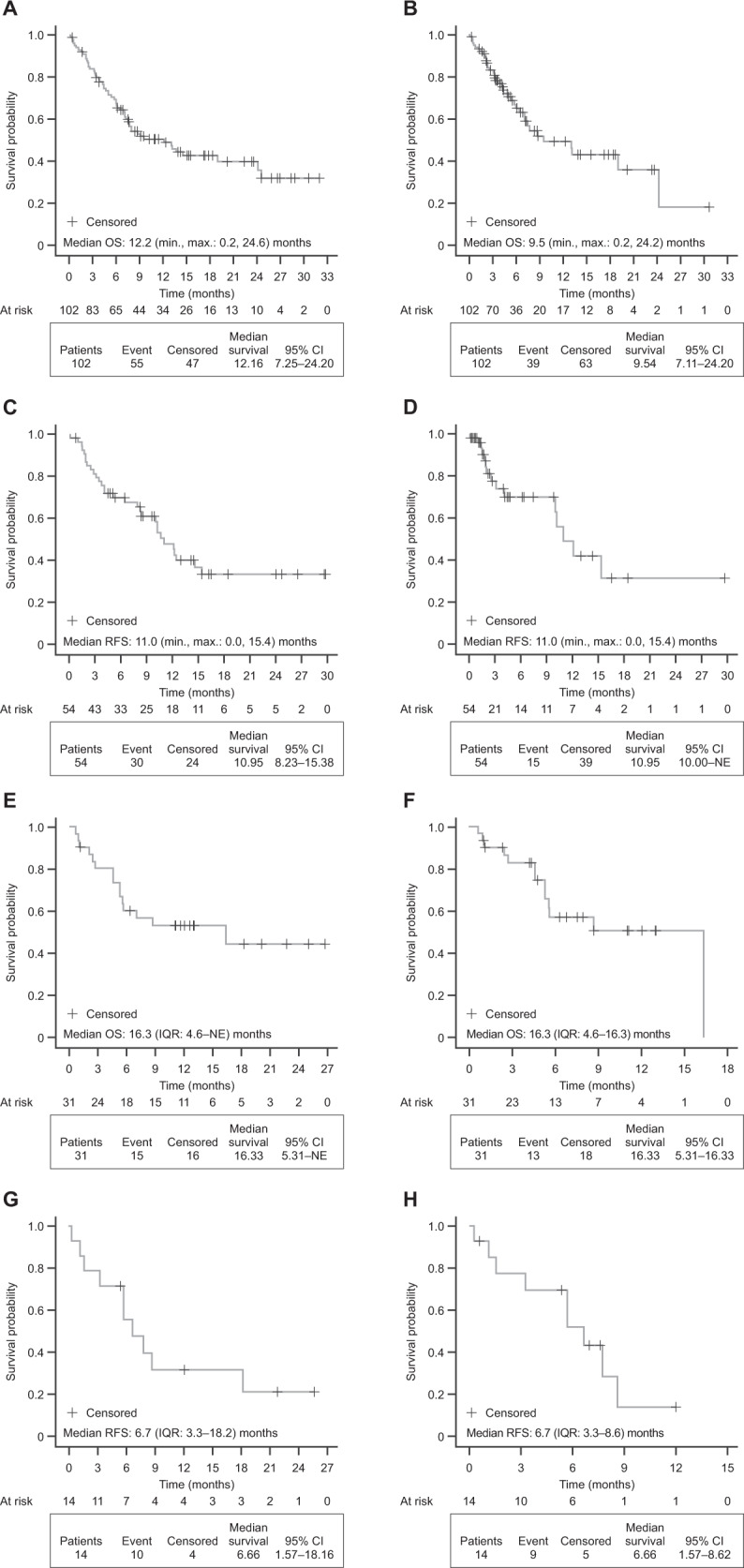
KM analysis of OS and RFS in the R/R Ph− and R/R Ph+ subgroups. **A** Time-to-event analysis for OS in the R/R Ph− subgroup. **B** OS with additional censoring at the time of HSCT in the R/R Ph− subgroup. **C** Time-to-event analysis for RFS in the R/R Ph− subgroup. **D** RFS with additional censoring at the time of HSCT in the R/R Ph− subgroup. **E** Time-to-event analysis for OS in the R/R Ph+ subgroup. **F** OS with additional censoring at the time of HSCT in the R/R Ph+ subgroup. **G** Time-to-event analysis for RFS in the R/R Ph+ subgroup. **H** RFS with additional censoring at the time of HSCT in the R/R Ph+ subgroup. In the R/R Ph− subgroup, four patients were excluded from the analysis owing to missing data. In the R/R Ph+ subgroup, three patients were excluded from the analysis because of missing data. For OS and RFS, patients were censored if they were alive at the end of the study or they were lost to follow-up. *CI* confidence interval, *HSCT* hematopoietic stem cell transplantation, *IQR* interquartile range, *max*. maximum, KM Kaplan–Meier, *min*. minimum, *NE* not estimable, *OS* overall survival, *Ph*− Philadelphia chromosome-negative, *Ph*+ Philadelphia chromosome-positive, *R/R* relapsed/refractory, *RFS* relapse-free survival.

In the Ph− subgroup, median (95% CI) OS was 12.2 (7.3–24.2) months (Fig. 3A; Supplementary Table 4), and 9.5 (7.1–24.2) months when censoring for HSCT (Fig. 3B). Median (95% CI) RFS was 11.0 (8.2–15.4) months with and without additional censoring at the time of HSCT (Fig. 3C, D; Supplementary Table 6). The KM estimate (95% CI) for RFS at 24 months was 33.1% (19.0–47.8%) overall (Supplementary Table 6), 35.0% in MRD responders, and 28.6% in MRD nonresponders (Supplementary Fig. 4). KM estimates for OS at 24 months were better among responders (CR/CRh/CRi or MRD) versus nonresponders, in patients with a prior HSCT versus no prior HSCT, and in patients with bone marrow blasts <50% versus ≥50% at blinatumomab initiation (Supplementary Fig. 2B). In the Ph+ subgroup, median (95% CI) OS was 16.3 (5.3–NE) months (Fig. 3E), and 16.3 (5.3–16.3) months with additional censoring at the time of HSCT (Fig. 3F). KM estimates for OS at 24 months were 56.6% in responders and 46.4% in nonresponders (Supplementary Fig. 2C). Median RFS was 6.7 months in analyses with or without additional censoring at the time of HSCT (Fig. 3G, H; Supplementary Table 6). The KM estimate (95% CI) for RFS at 24 months was 21.2% (4.0–47.3%) (Supplementary Table 6).

#### OS and RFS in R/R patients with DLI

In the Ph− subgroup of patients who received a DLI, four out of 13 died from any cause. At 12 months and 18 months, OS (95% CI) was 76.2% (42.7–91.7%) and 66.6% (33.1–86.1%), respectively. Of the nine patients with CR/CRh/CRi, one patient died from any cause and three patients relapsed. At 12 months and 18 months, RFS (95% CI) was 51.9% (16.4–78.8%).

### Outcomes following blinatumomab treatment

In the MRD+ group, 74 patients (68%) receiving blinatumomab proceeded to HSCT (Supplementary Table 7). Of these, 66% (*n* = 49) achieved an MRD response before transplant. In patients with an MRD response following blinatumomab who did not have any cytotoxic therapy (*n* = 43) between blinatumomab and transplant (Supplementary Table 7), the median (95% CI) time from achieving an MRD response to HSCT was 2.3 (1.6–9.1) months.

In the R/R group, of the Ph− patients who were in CR/CRh/CRi, 43 (41%) proceeded to HSCT (Supplementary Table 7), among whom 33 (77%) achieved CR/CRh/CRi before transplant. Of these 33 patients, 29 achieved CR/CRh/CRi and proceeded to HSCT without additional cytotoxic therapy. In this subgroup, median (95% CI) time from achieving CR/CRh/CRi to HSCT was 4.6 (2.2–NE) months.

Among the Ph+ patients in the R/R group who were in CR/CRh/CRi, 11 (32%) proceeded to HSCT following blinatumomab treatment (Supplementary Table 7). Of these, six (55%) achieved CR/CRh/CRi after blinatumomab initiation and all were treated with additional cytotoxic therapy before HSCT. Median time to HSCT was not reached.

### Nonrelapse mortality after HSCT

In the MRD+ group, mortality following HSCT was evaluable in 56 patients. Nonrelapse mortality (95% CI) at 12 months was 10.1% (4.3–23.8%; Table 2). In the Ph− and Ph+ subgroups, 12-month mortality (95% CI) was 9.0% (3.3–24.4%) and 18.1% (3.7–90.0%), respectively.

**Table 2:** Mortality after allogeneic HSCT by B-cell ALL diagnosis.

	MRD+			R/R B-cell ALL	
	All MRD+ (*n* = 56)	Ph− (*n* = 50)^a^	Ph+ (*n* = 6)	Ph−^b^ (*n* = 29)	Ph+^c^ (*n* = 6)
Mortality following HSCT, % (95% CI)^d^					
3 months	5.5 (1.9–16.3)	4.1 (1.1–15.2)	18.1 (3.7–90.0)	10.7 (3.4–34.0)	15.4 (2.8–84.6)
6 months	5.5 (1.9–16.3)	4.1 (1.1–15.2)	18.1 (3.7–90.0)	14.8 (5.8–37.4)	15.4 (2.8–84.6)
12 months	10.1 (4.3–23.8)	9.0 (3.3–24.4)	18.1 (3.7–90.0)	19.3 (9.3–40.4)	15.4 (2.9–84.6)
Events, ***n*** **(%)**					
Nonrelapse deaths	6 (11)	5 (10)	1 (16.7)	5 (17.2)	1 (16.7)
Relapse	11 (20)	11 (22)	0 (0.0)	8 (27.6)	1 (16.7)
Death due to undocumented relapse	3 (5)	3 (6)	0 (0.0)	2 (6.9)	0 (0.0)
Death due to unknown causes	1 (2)	1 (2)	0 (0.0)	0 (0.0)	0 (0.0)
Patients alive without relapse	34 (61)	29 (58)	5 (83.3)	14 (48.3)	4 (66.7)

^a^One patient excluded owing to missing data on date of event.

^b^Patients achieved CR/CRh/CRi following blinatumomab treatment and proceeded to HSCT without any intervening cytotoxic therapy.

^c^Patients achieved CR/CRh/CRi following blinatumomab treatment and were treated with other cytotoxic therapy and then proceeded to HSCT (all R/R Ph+ patients who proceeded to transplantation were treated with additional cytotoxic therapy following blinatumomab).

^d^Mortality not due to disease relapse is evaluated by the cumulative incidence function, with relapse and death due to undocumented relapse as competing events.

*B-cell ALL* B-cell acute lymphoblastic leukemia, *CI* confidence interval, *CR* complete remission with full recovery of peripheral blood counts, *CRh* complete remission with partial recovery of peripheral blood counts, *CRi* complete remission with incomplete recovery of peripheral blood counts, *HSCT* hematopoietic stem cell transplantation, *MRD*+ minimal residual disease-positive, *Ph*− Philadelphia chromosome-negative, *Ph*+ Philadelphia chromosome-positive, *R/R* relapsed/refractory.

All patients were treated with additional immunosuppressive therapy between blinatumomab and allogenic HSCT.

In the R/R group, mortality following HSCT was evaluable in 29 Ph− patients, and nonrelapse mortality (95% CI) at 12 months was 19.3% (9.3–40.4%). In the Ph+ subgroup, mortality following HSCT was evaluable in six patients, and nonrelapse mortality (95% CI) following HSCT at 12 months was 15.4% (2.9–84.6%) (Table 2).

## Discussion

The NEUF study describes the largest documented European cohort of patients treated with blinatumomab in real-world clinical practice reported to date. The real-world effectiveness of blinatumomab in adults with MRD+ or R/R ALL was comparable to that reported in other clinical and real-world studies [2, 3, 6, 9–15].

In the MRD+ group, over half of patients were in CR1 and 84% of patients with an evaluable MRD achieved an overall MRD response within the first two cycles of blinatumomab therapy. Moreover, the MRD+ group included more than two-thirds of patients with persistent MRD prior to initiating blinatumomab therapy. The overall results in the MRD+ group align with previously reported efficacy data from clinical and real-world studies. In the Phase 2 clinical trial, 78% of patients achieved a complete MRD response [3]. In the FRENCH-CYTO study, 89% of the MRD+ patients achieved a complete MRD response following blinatumomab [13]. Crucially, the NEUF study shows that survival outcomes were improved in patients who did versus did not have an MRD response, and in patients in the CR1 versus CR2+ subgroup, in line with earlier findings from the Phase 2 trial [6]. Nonrelapse mortality at 12 months was 10% in the MRD+ group.

In the R/R Ph− subgroup, over half of patients achieved CR/CRh/CRi and over one-third proceeded to HSCT, in line with the results of the Phase 3 trial in which nearly half of patients had also received blinatumomab in their first salvage [9, 10]. The transplant realization rate was considerably higher in patients in the NEUF study than in the Phase 3 clinical trial. The relatively short median interval from achieving CR/CRh/CRi to HSCT of 4.6 months among patients who did not receive other cytotoxic therapy indicates that blinatumomab is being used as a bridge to transplantation. Importantly, patients who had an HSCT before blinatumomab initiation as well as patients who achieved CR/CRh/CRi and/or an MRD response following blinatumomab treatment had an improved survival result compared with patients who did not. Moreover, given the good results when censoring for HSCT, blinatumomab could be considered to be a valuable option also for older patients unfit for transplantation. Despite some differences in patient characteristics and outcomes, the data of the NEUF study align overall with the literature [13–15].

In the R/R Ph+ subgroup, over 40% of patients achieved CR/CRh/CRi within two cycles of blinatumomab initiation and over two-thirds of these patients with available MRD data achieved an MRD response. Most patients in the R/R Ph+ subgroup had at least one prior salvage therapy. Over one-third of patients achieved CR/CRh/CRi and proceeded to allogeneic HSCT without further therapy. This was similar to results reported in a Phase 2 trial of patients with R/R Ph+ , in which 36% of patients achieved CR/CRh/CRi within two cycles of blinatumomab initiation and 88% of these patients achieved an MRD response [12]. As expected, the Ph+ subgroup had slightly worse outcomes than the Ph− subgroup. Patients with Ph+ B-cell ALL are considered to be high risk owing to their cytogenetic profile and the limited MRD response to treatment [22, 23]. With regard to the improvement of the MRD response induced by blinatumomab in patients with Ph+ B-cell ALL, its efficacy in patients who were newly diagnosed with Ph+ B-cell ALL was reported in the D-ALBA study [23], in which 60% of patients achieved a molecular response following treatment with dasatinib and two cycles of blinatumomab. Molecular response rates further increased with additional cycles of blinatumomab. As proposed by Puzzolo et al, the host immune system may contribute to this effect of blinatumomab in this setting [24]. This may explain the encouraging results obtained in patients treated with DLI.

In a proportion of patients with extramedullary involvement included in the NEUF study, blinatumomab elicited a response regardless of Ph status, and this response was consistent with the cytomorphological response of each Ph− or Ph+ subgroup; this is one of the first reports on the effect of blinatumomab in this subset of patients. Overall, these results highlight the real-world effectiveness of blinatumomab in different subgroups of patients with B-cell ALL.

The limitations of this study merit consideration and have been detailed previously [16]. Individuals enrolled in an EAP may not be representative of the wider population of patients with B-cell ALL receiving blinatumomab treatment. An additional limitation of the study was a potential patient selection bias given that only sites with the largest proportion of expanded access requests across the selected countries were invited to participate in the NEUF study. Of those, 51 sites participated. Finally, a small proportion of patients in the MRD+ group demonstrated bone marrow blasts >5%; this may indicate that clinicians enrolled patients with an advanced disease or that the patient’s disease had progressed by the time they received blinatumomab.

In conclusion, these results from the largest European real-word cohort of adults with B-cell ALL are consistent with data from clinical and real-world studies, confirming the effectiveness of blinatumomab in both the MRD+ and R/R settings.

## Supplementary information

Supplementary material

Supplementary Fig 1

Supplementary Fig 2

Supplementary Fig 3

Supplementary Fig 4

Reproducibility checklist

## Data Availability

Data that support the results of this study are available from the corresponding author upon reasonable request. Qualified researchers may request data from Amgen studies. Details are available at the following: http://www.amgen.com/datasharing.
