# Supplementary material for: Real-world use of blinatumomab in adult patients with B-cell acute lymphoblastic leukemia in clinical practice: results from the NEUF study
Source: Blood Cancer J. 2023 Jan 4;13(1):2. doi: 10.1038/s41408-022-00766-7 (PMC9813344; doi:10.1038/s41408-022-00766-7)
Supplement: Supplementary file 1 — Supplementary material [file 41408_2022_766_MOESM1_ESM.docx]

**Supplementary material**

**Supplementary Fig. 1.** Distribution of patients with **A** MRD+ and **B** R/R Ph+ B-cell ALL by specific tyrosine kinase inhibitors used as frontline therapy or salvage therapies. *B-cell ALL* B-cell acute lymphoblastic leukemia, *MRD+* minimal residual disease-positive, *Ph+* Philadelphia chromosome-positive, *R/R* relapse/refractory.

**Supplementary Fig. 2.** OS at 24 months in all patients and by subgroup (KM estimates). **A** MRD+. **B** R/R Ph−. **C** R/R Ph+. *CI* confidence interval, *CR* complete remission with full recovery of peripheral blood counts, *CRh* complete remission with partial recovery of peripheral blood counts, *CRi* complete remission with incomplete recovery of peripheral blood counts, CR1 defined patients who reached a complete remission after first induction therapy; CR2+ defined patients who reached complete remission after one or more salvage therapies for relapsed or refractory disease. *HSCT* hematopoietic stem cell transplantation, *KM* Kaplan–Meier, *MRD* minimal residual disease, *MRD+* MRD-positive, *NE* not estimable, *OS* overall survival, *Ph−* Philadelphia chromosome-negative, *Ph+* Philadelphia chromosome-positive, *R/R* relapsed/refractory*.*

**Supplementary Fig. 3.** DFS at 24 months in MRD+ patients by subgroup (KM estimates). One patient was excluded from the analysis owing to missing data (dates). *CI* confidence interval, CR1 defined patients who reached a complete remission after first induction therapy; CR2+ defined patients who reached complete remission after one or more salvage therapies for relapsed or refractory disease, *DFS* disease-free survival, *HSCT* hematopoietic stem cell transplantation, *KM* Kaplan–Meier, *MRD* minimal residual disease, *MRD+* MRD-positive, *Ph−* Philadelphia chromosome-negative, *Ph+* Philadelphia chromosome-positive.

**Supplementary Fig. 4.** RFS at 24 months in the R/R Ph− by subgroup (KM estimates). RFS was defined as the interval from the time of achieving CR/CRh/CRi (best response within the first two cycles) until the date of relapse; M2 marrow, death (whichever occurred first). *CI* confidence interval, *CR* complete remission with full recovery of peripheral blood counts, *CRh* complete remission with partial recovery of peripheral blood counts, *CRi* complete remission with incomplete recovery of peripheral blood counts, *HSCT* hematopoietic stem cell transplant, *KM* Kaplan–Meier, *MRD* minimal residual disease, *Ph−* Philadelphia chromosome-negative, *RFS* relapse-free survival, *R/R* relapsed/refractory.

**Supplementary Table 1.** Overview of techniques used to assess MRD before blinatumomab initiation in patients with Ph+ B-cell ALL (MRD+ and R/R groups) according to cytogenetic profile.

|  | **MRD+ Ph+ (n=26)** | | | **R/R Ph+ (n=34)** | | |
| --- | --- | --- | --- | --- | --- | --- |
| **Cytogenetics** | **Patients,**  **n (%)** | **Technique(s) used for MRD marker testing** | **Patients,**  **n (%)** | **Patients,  n (%)** | **Technique(s) used for MRD marker testing** | **Patients,**  **n (%)** |
| Normal karyotype | 2 (7.7) | Gene fusion | 2 (100) | 2 (5.9) | Gene fusion | 2 |
| High hyperdiploidy | 2 (7.7) | Gene fusion | 2 (100) | 1 (2.9) | Gene fusion | 1 |
| Hypodiploidy | 2 (7.7) | Gene fusion | 2 (100) | 0 (0.0) | _ | _ |
| t(9;22)(q34;q11.2)/BCR-ABL1 | 26 (100.0) | Gene fusion | 20 (76.9) | 34 (100.0) | Gene fusion | 19 (55.9) |
|  |  | Gene fusion and flow cytometry | 3 (11.5) |  | Gene fusion and flow cytometry | 7 (20.6) |
|  |  | Gene fusion and IgH/TCR | 0 |  | Gene fusion and IgH/TCR | 2 (5.9) |
|  |  | Flow cytometry | 3 (11.5) |  | Flow cytometry | 2 (5.9) |
|  |  | IgH/TCR | 0 |  | IgH/TCR | 1 (2.9) |
|  |  |  |  |  | No testing documented | 3 (8.8) |
| Other | 2 (7.7) | Gene fusion | 2 (100) | 3 (8.8) | Gene fusion | 1 (33.3) |
|  |  | Flow cytometry | 0 |  | Flow cytometry | 1 (33.3) |
|  |  | Flow cytometry and gene fusion | 0 |  | Flow cytometry and gene fusion | 1 (33.3) |

Patients may have undergone multiple testing procedures. *B-cell* ALL B-cell acute lymphoblastic leukemia, *IgH* immunoglobulin heavy chain*, MRD+* minimal residual disease-positive, *Ph+* Philadelphia chromosome-positive, *R/R* relapsed/refractory, *TCR* T cell receptor gene rearrangement.

## Supplementary Table 2. Concurrent use of tyrosine kinase inhibitors during blinatumomab treatment by subgroup.

| **Tyrosine kinase inhibitors, *n* (%)** | **MRD+** | **R/R** | |
| --- | --- | --- | --- |
|  | **Ph−/ Ph+^a^**  **(*n* = 109)** | **Ph−**  **(*n* = 106)** | **Ph+**  **(*n* = 34)** |
| Any  Missing  Imatinib  Dasatinib  Nilotinib  Bosutinib  Ponatinib | 13 (12.3)  3 (NA)  1 (0.9)  8 (7.5)  0 (0.0)  0 (0.0)  4 (3.8) | 1 (0.9)  0 (NA)  0 (0.0)  1 (0.9)  1 (0.9)  0 (0.0)  0 (0.0) | 14 (41.2)  0 (NA)  1 (2.9)  6 (17.6)  2 (5.9)  1 (2.9)  6 (17.6) |

^a^One Ph− patient received a tyrosine kinase inhibitor.

*MRD+* minimal residual disease-positive, *NA* not applicable, *Ph−* Philadelphia chromosome-negative, *Ph+* Philadelphia chromosome-positive, *R/R* relapsed/refractory.

## Supplementary Table 3. Comedications with blinatumomab by subgroup.

| **Comedications with blinatumomab, *n* (%)** | **MRD+** | | | **R/R B-cell ALL** | |
| --- | --- | --- | --- | --- | --- |
|  | **All** (*n* = 109) | **Ph−**  **(*n* = 83)** | **Ph+**  **(*n* = 26)** | **Ph−**  **(*n* = 106)** | **Ph+**  **(*n* = 34)** |
| Donor lymphocyte infusion  Missing  Chemotherapy  Missing | 4 (3.8)  3 (NA)  7 (6.5)  2 (NA) | 2 (2.5)  3 (NA)  6 (7.3)  1 (NA) | 2 (7.7)  0 (NA)  1 (4.0)  1 (NA) | 13 (12.9)  5 (NA)  10 (9.4)  0 (NA) | 0 (0.0)  0 (NA)  5 (14.7)  0 (NA) |

*B-cell ALL* B-cell acute lymphoblastic leukemia, *MRD+* minimal residual disease-positive, *NA* not applicable, *Ph−* Philadelphia chromosome-negative, *Ph+* Philadelphia chromosome-positive, *R/R* relapsed/refractory.

**Supplementary Table 4.** OS by subgroup.

|  | **MRD+** | | | **R/R** | |
| --- | --- | --- | --- | --- | --- |
|  | **All MRD+^a^ (*n* = 109)^b^** | **Ph−^c^ (*n* = 83)^b^** | **Ph+^d^ (*n* = 26)** | **Ph**–**^e^ (*n* = 106)^f^** | **Ph+^g^ (*n* = 34)^h^** |
| Patients, *n* (%)  Event (death)  Censored (alive at end of study, lost to follow-up) | 33 (30.3)  74 (67.9) | 28 (33.7)  53 (63.9) | 5 (19.2)  21 (80.8) | 55 (51.9)  47 (44.3) | 15 (44.1)  16 (47.1) |
| KM estimate, % (95% CI)  At 1 month  At 3 months  At 6 months  At 12 months  At 18 months  At 24 months | 98.1 (92.7–99.5)  96.3 (90.3–98.6)  92.4 (85.4–96.1)  77.9 (68.0–85.0)  66.9 (55.6–75.9)  64.7 (52.8–74.2) | 98.8 (91.6–99.8)  96.3 (89.0–98.8)  95.1 (87.4–98.1)  76.4 (64.7–84.7)  62.4 (49.3–73.1)  62.4 (49.3–73.1) | 96.2 (75.7–99.4)  96.2 (75.7–99.4)  83.6 (62.0–93.5)  83.6 (62.0–93.5)  83.6 (62.0–93.5)  71.7 (38.6–89.0) | 94.1 (87.3–97.3)  84.0 (75.2–89.9)  67.5 (57.3–75.8)  50.6 (40.0–60.2)  42.8 (32.1–53.2)  40.0 (28.7–51.0) | 93.5 (76.6–98.3)  80.3 (61.3–90.6)  60.2 (40.7–75.1)  53.1 (34.0–69.1)  44.3 (22.5–64.1)  44.3 (22.5–64.1) |

^a^Median OS not reached over a median follow-up (range) of 18.5 (1.8–34.8) months.

^b^Three patients were excluded from the analysis due to missing date data.

^c^Median OS was not reached over a median (range) follow-up time of 18.8 (5.1–34.8) months.

^d^Median OS was not reached over a median (range) follow-up time of 16.5 (1.8–31.6) months.

^e^Median (95% CI) OS was 12.2 (7.3–24.2) months over a median (range) follow-up time of 17.3 (0.4–32.1) months.

^f^Two patients were excluded from the analysis owing to missing data (dates).

^g^Median (95% CI) OS was 16.3 (5.3–NE) months over a median (range) follow-up time of 13.0 (1.1–26.7) months.

^h^Four patients were excluded from the analysis due to missing date data.

*CI* confidence interval, *KM* Kaplan–Meier, *MRD+* minimal residual disease-positive, *NE* not estimated, *OS* overall survival, *Ph−* Philadelphia chromosome-negative, *Ph+* Philadelphia chromosome-positive, *R/R* relapsed/refractory.

**Supplementary Table 5.** Landmark analysis of OS in selected subgroups**.**

|  | **Landmark analyses** | | | | | |
| --- | --- | --- | --- | --- | --- | --- |
|  | **MRD+ (*n* = 103)** | | **MRD+ Ph−  (*n* = 79)** | | **R/R Ph−  (*n* = 83)** | |
|  | **Responders^a^ (*n* = 82)^b^** | **Nonresponders^c^ (*n* = 21)^d^** | **Responders^e^ (*n* = 66)** | **Nonresponders^f^ (n = 13)** | **Responders^e^ (*n* = 50)** | **Nonresponders^f^ (*n* = 33)** |
| Median OS (95% CI) | NE | NE | NE | 14.2 (6.8–NE) | 21.8 (10.4–NE) | 5.0 (3.7–21.4) |
| Median follow-up (range) | 16.5 (2.4–32.0) | 13.5 (0.6–28.6) | 16.5 (2.4–32.0) | 14.0 (3.6–28.6) | 14.5 (1.0–29.3) | 14.5 (7.5–24.2) |
| KM estimate for survival at 24 months (95% CI) | 64.2  (49.6–75.6) | 58.3  (24.2–81.4) | 62.8  (46.8–75.2) | 47.6  (13.7–75.8) | 49.0  (28.3–66.9) | 20.8  (4.5–45.2) |

^a^MRD response within the first 84 days following blinatumomab initiation.

^b^Includes 16 Ph+ patients.

^c^No MRD response within the first 84 days following blinatumomab initiation.

^d^Includes eight Ph+ patients.

^e^CR/CRh/CRi within the first 84 days following blinatumomab initiation.

^d^No CR/CRh/CRi within the first 84 days following blinatumomab initiation.

*CI* confidence interval, *CR* complete remission with full recovery of peripheral blood counts, *CRh* complete remission with partial recovery of peripheral blood counts, *CRi* complete remission with incomplete recovery of peripheral blood counts, *KM* Kaplan–Meier, *MRD* minimal residual disease, *MRD+* MRD-positive, *NE* not estimated, *OS* overall survival, *Ph−* Philadelphia chromosome-negative, *Ph+* Philadelphia chromosome-positive, *R/R* relapsed/refractory.

**Supplementary Table 6.** RFS and DFS by subgroup.

|  | **DFS** | | | **RFS** | |
| --- | --- | --- | --- | --- | --- |
|  | **MRD+** | | | **R/R Ph−**^e^ **(*n* = 54)** | **R/R Ph+^f^ (*n* = 14)** |
|  | **All MRD+^a^  (*n* = 109)^b^** | **Ph−^c^ (*n* = 83)** | **Ph+^d^ (*n* = 26)** |  |  |
| Median survival (95% CI) | 27.6 (13.0–NE) | 25.7 (11.7–NE) | Not reached | 11.0 (8.2–15.4) | 6.7 (1.6–18.2) |
| Patients, *n* (%)  Event (death or relapse)  Censored (alive at end of study, lost to follow-up) | 46 (42.2)  62 (56.9) | 37 (44.6)  45 (54.2) | 9 (34.6)  17 (65.4) | 30 (55.6)  24 (44.4) | 10 (71.4)  4 (28.6) |
| KM estimate, % (95% CI)  At 1 month  At 3 months  At 6 months  At 12 months  At 18 months  At 24 months | 98.1 (92.8–99.5)  92.6 (85.7–96.2)  84.0 (75.5–89.7)  62.2 (51.9–71.0)  55.1 (44.1–64.7)  55.1 (44.1–64.7) | 98.8 (91.7–99.8)  93.9 (86.0–97.4)  86.5 (77.0–92.3)  61.2 (49.3–71.2)  54.1 (41.7–65.0)  54.1 (41.7–65.0) | 96.2 (75.7–99.4)  88.1 (67.6–96.0)  75.7 (53.7–88.3)  65.9 (42.9–81.5)  57.7 (32.4–76.4)  57.7 (32.4–76.4) | 96.3 (85.9–99.1)  81.2 (67.8–89.4)  69.7 (55.3–80.2)  47.6 (32.4–61.3)  33.1 (19.0–47.8)  33.1 (19.0–47.8) | 92.9 (59.1–99.0)  78.6 (47.2–92.5)  55.6 (26.4–77.2)  31.7 (9.9–56.5)  31.7 (9.9–56.5)  21.2 (4.0–47.3) |
| Median follow-up (range), months | 18.3 (1.8–34.8) | 18.6 (5.1–34.8) | 16.2 (1.8–28.5) | 16.2 (0.7–29.7) | 21.7 (5.4–25.6) |

^a^Median (95% CI) DFS was 27.6 (13.0–NE) months over a median (range) follow-up time of 18.3 (1.8–34.8) months.
^b^One patient was excluded from the analyses owing to missing data (dates).
^c^Median (95% CI) DFS was 25.7 (11.7–NE) months over a median (range) follow-up time of 18.6 (5.1–34.8) months.
^d^Median (95% CI) DFS was not reached over a median (range) follow-up time of 16.2 (1.8–28.5) months.
^e^Median (95% CI) RFS was 11.0 (8.2–15.4) months over a median (range) follow-up time of 16.2 (0.7–29.7) months.
^f^Median (95% CI) RFS was 6.7 (1.6–18.2) months over a median (range) follow-up time of 21.7 (5.4–25.6) months.

*CI* confidence interval, *DFS* disease-free survival, *KM* Kaplan–Meier, *MRD+* minimal residual disease-positive, *NE* not estimable, *OS* overall survival, *Ph−* Philadelphia chromosome-negative, *Ph+* Philadelphia chromosome-positive, *R/R* relapsed/refractory, *RFS* relapse-free survival.

**Supplementary Table 7.** Therapy/HSCT following blinatumomab treatment.

|  | | **MRD+** | | | **R/R** | |
| --- | --- | --- | --- | --- | --- | --- |
|  | | **All**  **(*n* = 109)** | **Ph−**  **(*n* = 83)** | **Ph+**  **(*n* = 26)** | **Ph−**  **(*n* = 106)** | **Ph+**  **(*n* = 34)** |
| Median number of cycles of blinatumomab started  Patients without HSCT after blinatumomab  Patients undergoing HSCT at any time following blinatumomab initiation | | 2  2 | 3  2 | 2  2 | 2  2 | 2  3 |
| HSCT at any time following blinatumomab initiation | |  |  |  |  |  |
| Total number of patients | | 74 (67.9) | 58 (69.9) | 16 (61.5) | 43 (40.6) | 11 (32.4) |
| Patients with CR/CRh/CRi who proceeded to allogeneic HSCT | | – | – | – | 33 (76.7) | 6 (54.5) |
| Patients with MRD response who proceeded to allogeneic HSCT | | 49 (66.2) | 44 (75.9) | 5 (31.3) | – | – |
| Patients with CR/CRh/CRi who proceeded to allogeneic HSCT without additional cytotoxic therapy | | – | – | – | 29 (67.4) | 0 (0.0) |
| Patients with MRD response who proceeded to allogeneic HSCT without additional cytotoxic therapy | | 43 (58.1) | 41 70.7) | 2 (12.5) | – | – |
| Conditioning regimen | |  |  |  |  |  |
| Myeloablative conditioning | | 59 (84.3) | 49 (89.1) | 10 (66.7) | 28 (70.0) | 8 (72.7) |
| Reduced intensity conditioning | | 12 (17.1) | 7 (12.7) | 5 (33.3) | 12 (30.0) | 4 (36.4) |
| Unknown | | 4 (–) | 3 (–) | 1 (–) | 3 (–) | 0 (–) |
| Graft failure | |  |  |  |  |  |
| Yes | | 5 (7.1) | 5 (9.1) | 0 (0.0) | 2 (4.7) | 2 (18.2) |
| Missing | | 4 (–) | 3 (–) | 1 (–) | 0 (–) | 0 (–) |
| Blinatumomab re-treatment, *n* | | 9 | 7 | 2 | 7 | NA |
| **Proceeded to other treatment following blinatumomab initiation** | | | | | | |
| **HSCT following new treatment: (yes/no)** | **Response^c^ before new treatment: (achieved/not achieved)** |  | | | | |
| *Proceeded to tyrosine kinase inhibitors, n* | | 3 | 0 | 3 | 0 | 7 |
| No^a^ | Achieved | 0 (0.0) | 0 (0.0) | 0 (0.0) | 0 (0.0) | 3 (42.9) |
|  | Not achieved | 3 (100.0) | 0 (0.0) | 3 (100.0) | 0 (0.0) | 2 (28.6) |
| Yes^b^ | Achieved | 0 (0.0) | 0 (0.0) | 0 (0.0) | 0 (0.0) | 1 (14.3) |
|  | Not achieved | 0 (0.0) | 0 (0.0) | 0 (0.0) | 0 (0.0) | 1 (14.3) |
| **Total** | Achieved | 0 (0.0) | 0 (0.0) | 0 (0.0) | 0 (0.0) | 4 (57.1) |
|  | Not achieved | 3 (100.0) | 0 (0.0) | 3 (100.0) | 0 (0.0) | 3 (42.9) |
| *Proceeded to chemotherapy, n* | | 26 | 22 | 4 | 36 | 7 |
| No | Achieved | 13 (50.0) | 11 (50.0) | 2 (50.0) | 14 (38.9) | 2 (28.9) |
|  | Not achieved | 6 (23.1) | 5 (22.7) | 1 (25.0) | 14 (38.9) | 4 (57.1) |
| Yes | Achieved | 2 (7.7) | 2 (9.1) | 0 (0.0) | 2 (5.6) | 0 (0.0) |
|  | Not achieved | 5 (19.2) | 4 (18.2) | 1 (25.0) | 6 (16.7) | 1 (14.3) |
| **Total** | Achieved | 15 (57.7) | 13 (59.1) | 2 (50.0) | 16 (44.4) | 2 (28.9) |
|  | Not achieved | 11 (42.3) | 9 (40.9) | 2 (50.0) | 20 (55.6) | 5 (71.4) |
| *Proceeded to inotuzumab, n* | | 12 | 11 | 1 | 17 | 2 |
| No | Achieved | 6 (50.0) | 6 (54.5) | 0 (0.0) | 6 (35.3) | 2 (100.0) |
|  | Not achieved | 2 (16.7) | 1(9.1) | 1 (100.0) | 6 (35.3) | 0 (0.0) |
| Yes | Achieved | 2 (16.7) | 2 (18.2) | 0 (0.0) | 1 (5.9) | 0 (0.0) |
|  | Not achieved | 2 (16.7) | 2 (18.2) | 0 (0.0) | 4 (23.5) | 0 (0.0) |
| **Total** | Achieved | 8 (66.7) | 8 (72.7) | 0 (0.0) | 7 (41.2) | 2 (100.0) |
|  | Not achieved | 4 (33.3) | 3 (27.3) | 1 (100.0) | 10 (58.8) | 0 (0.0) |

^a^Did not proceed to HSCT. ^b^Proceeded to HSCT. ^c^Response was measured in the Ph− subgroups as CR/CRh/CRi; response was measured as MRD in the MRD+ group.

All data are *n* (%) unless otherwise stated.

Analysis was neither restricted by the number of blinatumomab cycles nor whether a response occurred.

*CR* complete remission with full recovery of peripheral blood counts, *CRh* complete remission with partial recovery of peripheral blood counts, *CRi* complete remission with incomplete recovery of peripheral blood counts, *HSCT* hematopoietic stem cell transplantation, *MRD* minimal residual disease, *MRD+* MRD-positive, *NA* not available, *Ph−* Philadelphia chromosome-negative, *Ph+* Philadelphia chromosome-positive, *R/R* relapsed/refractory*.*
