# Supplementary figures and images for: Real-world use of blinatumomab in adult patients with B-cell acute lymphoblastic leukemia in clinical practice: results from the NEUF study
Source: Blood Cancer J. 2023 Jan 4;13(1):2. doi: 10.1038/s41408-022-00766-7 (PMC9813344; doi:10.1038/s41408-022-00766-7)

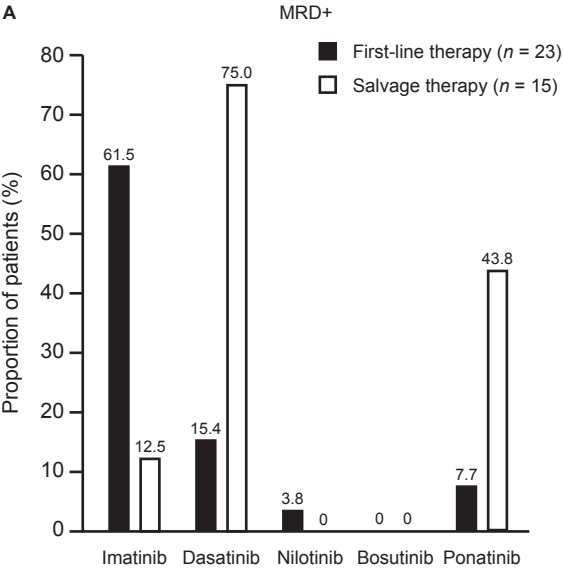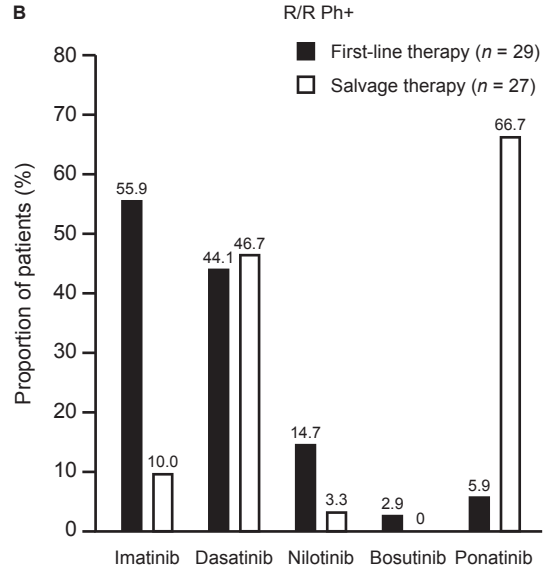

Supplement: Supplementary file 2 — Supplementary Fig 1 [file 41408_2022_766_MOESM2_ESM.pdf]

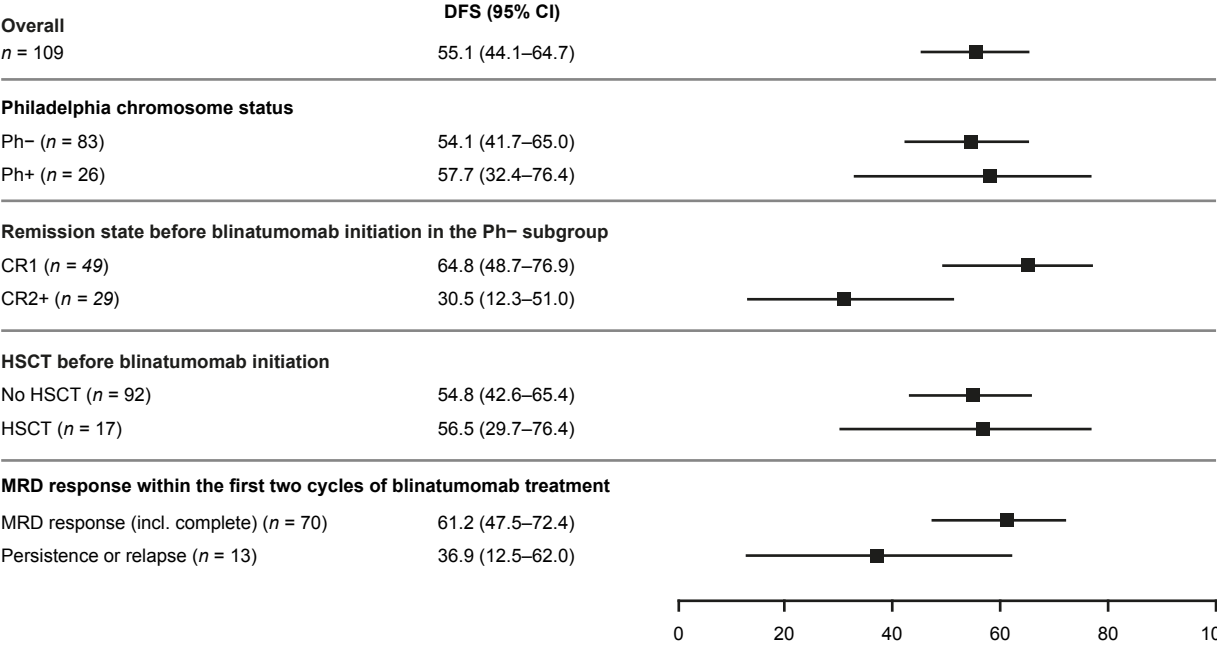

Supplement: Supplementary file 4 — Supplementary Fig 3 [file 41408_2022_766_MOESM4_ESM.pdf]

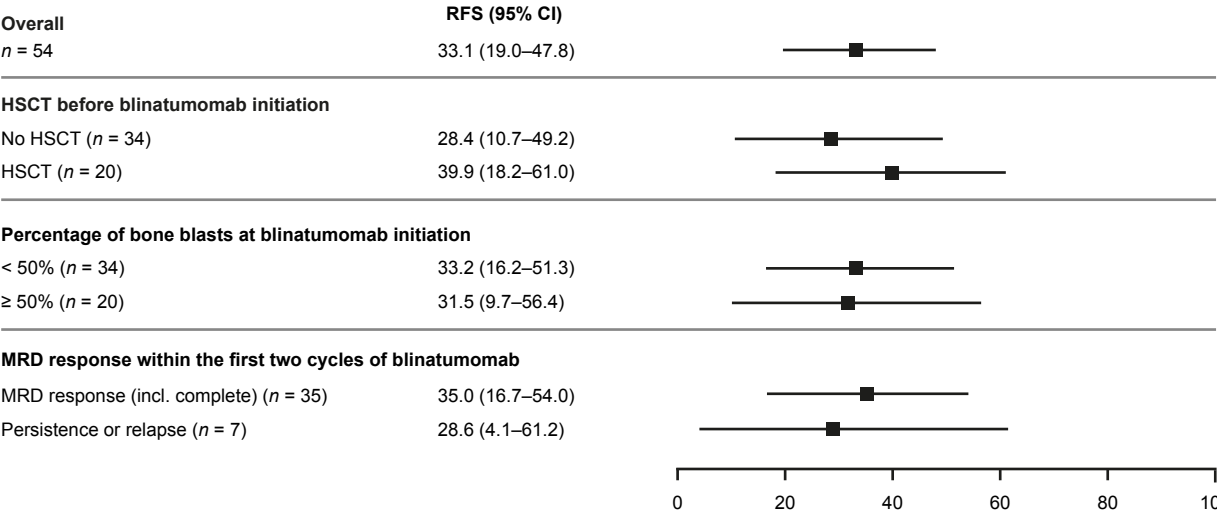

Supplement: Supplementary file 5 — Supplementary Fig 4 [file 41408_2022_766_MOESM5_ESM.pdf]
