# Supplementary material for: Real-world use of blinatumomab in adult patients with B-cell acute lymphoblastic leukemia in clinical practice: results from the NEUF study
Source: Blood Cancer J. 2023 Jan 4;13(1):2. doi: 10.1038/s41408-022-00766-7 (PMC9813344; doi:10.1038/s41408-022-00766-7)
Supplement: Supplementary file 3 — Supplementary Fig 2 [file 41408_2022_766_MOESM3_ESM.pdf]

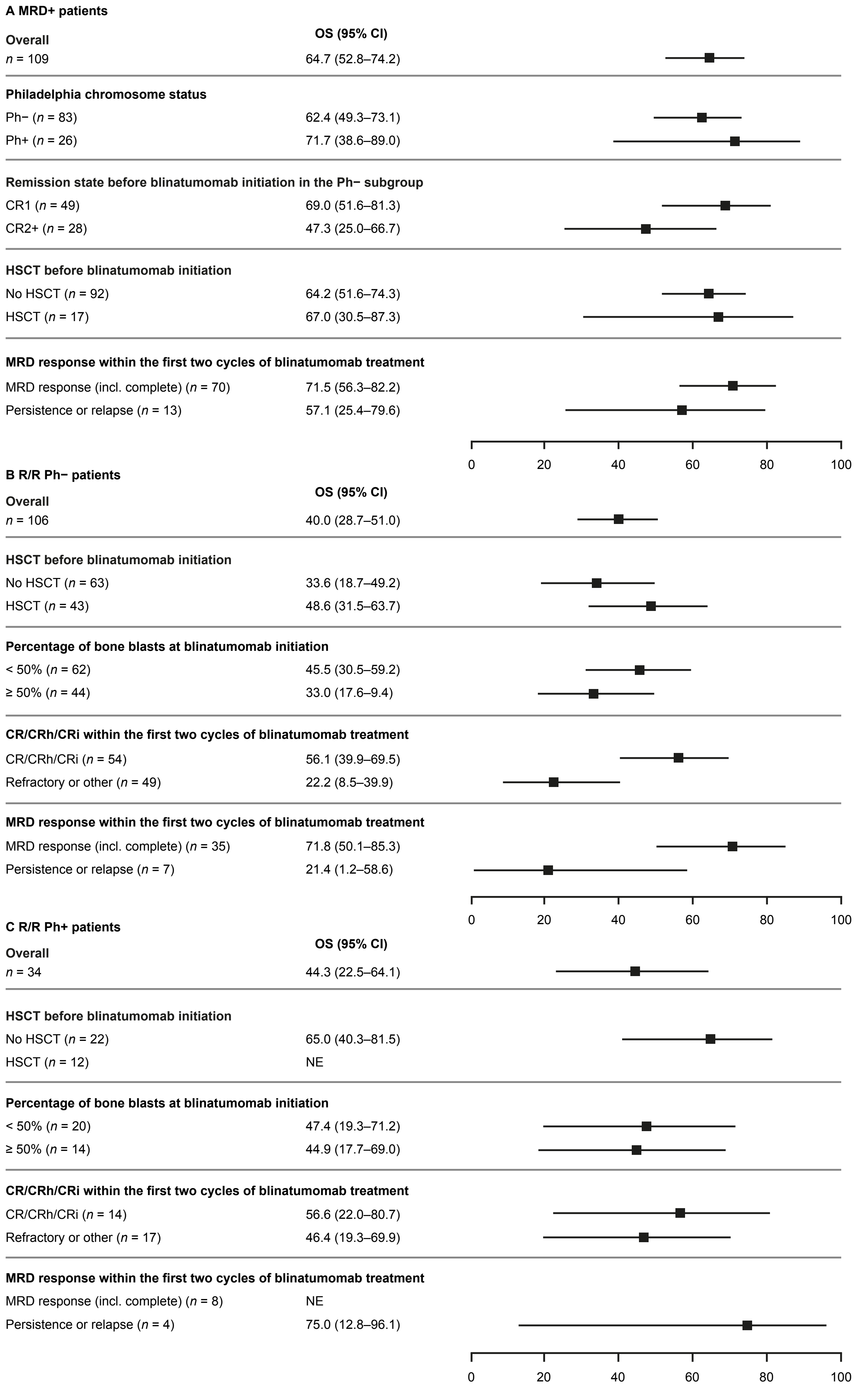
A MRD+ patients
OS (95% CI)
Overall
n = 109
64.7 (52.8–74.2)
Philadelphia chromosome status
Ph− (n = 83)
62.4 (49.3–73.1)
Ph+ (n = 26)
71.7 (38.6–89.0)
Remission state before blinatumomab initiation in the Ph− subgroup
CR1 (n = 49)
69.0 (51.6–81.3)
CR2+ (n = 28)
47.3 (25.0–66.7)
HSCT before blinatumomab initiation
No HSCT (n = 92)
64.2 (51.6–74.3)
HSCT (n = 17)
67.0 (30.5–87.3)
MRD response within the first two cycles of blinatumomab treatment
MRD response (incl. complete) (n = 70)
71.5 (56.3–82.2)
Persistence or relapse (n = 13)
57.1 (25.4–79.6)
0
20
40
60
80
100
B R/R Ph− patients
OS (95% CI)
Overall
n = 106
40.0 (28.7–51.0)
HSCT before blinatumomab initiation
No HSCT (n = 63)
33.6 (18.7–49.2)
HSCT (n = 43)
48.6 (31.5–63.7)
Percentage of bone blasts at blinatumomab initiation
< 50% (n = 62)
45.5 (30.5–59.2)
≥ 50% (n = 44)
33.0 (17.6–9.4)
CR/CRh/CRi within the first two cycles of blinatumomab treatment
CR/CRh/CRi (n = 54)
56.1 (39.9–69.5)
Refractory or other (n = 49)
22.2 (8.5–39.9)
MRD response within the first two cycles of blinatumomab treatment
MRD response (incl. complete) (n = 35)
71.8 (50.1–85.3)
Persistence or relapse (n = 7)
21.4 (1.2–58.6)
0
20
40
60
80
100
C R/R Ph+ patients
OS (95% CI)
Overall
n = 34
44.3 (22.5–64.1)
HSCT before blinatumomab initiation
No HSCT (n = 22)
65.0 (40.3–81.5)
HSCT (n = 12)
NE
Percentage of bone blasts at blinatumomab initiation
< 50% (n = 20)
47.4 (19.3–71.2)
≥ 50% (n = 14)
44.9 (17.7–69.0)
CR/CRh/CRi within the first two cycles of blinatumomab treatment
CR/CRh/CRi (n = 14)
56.6 (22.0–80.7)
Refractory or other (n = 17)
46.4 (19.3–69.9)
MRD response within the first two cycles of blinatumomab treatment
MRD response (incl. complete) (n = 8)
NE
Persistence or relapse (n = 4)
75.0 (12.8–96.1)
0
20
40
60
80
100
